# John Beddoe

**Published:** 1911-09

**Authors:** 


### Obituary.

### JOHN BEDDOE, M.D., LL.D., F.R.C.P., F.R.S.

It was only in our last volume (1910, page 359) that we gave a review of Dr. Beddoe's life as gathered from his own *Memories of Eighty Years* (Arrowsmith). He is no longer able to write his own biography, but who could write this better than himself in the life-story he has given us? It becomes our duty to record our appreciation of the work he has done during his eighty-four years, and to express our regret that the record is now closed. He has been called a veteran British anthropologist, and as such his reputation is world-wide as an erudite writer and a brilliant authority on all questions of ethnology and anthropology. But it is more particularly as a physician, and his work in the medical field, to which we would now refer. He was Physician to the Bristol Royal Infirmary from 1862 to 1873, when he resigned his office in order that he might have more leisure for his favourite scientific pursuits. For many years he was one of the leading physicians of the district, and it was a cause of great regret to a large circle of medical and other friends and patients when he retired from his work here and made his home at Bradford-on-Avon, where he died on July 19th, his funeral taking place at Edinburgh on July 22nd, 1911.

He held several other medical appointments in Bristol. The Hospital for Sick Women and Children, the Dispensary in Castle Green, and many other institutions claimed him from time to time as physician or consulting physician, and he was Physician to the Visiting Justices for Gloucestershire. Bristolians have shown their regard for his personality and appreciation of his work in many ways. A casket presented to him when he retired from Clifton bore the inscription : " A man peculiarly beloved by his brethren : a distinguished investigator of the human race." It will be of some interest to remind our readers that this artistic ebony and bronze casket was greatly the design of our former editor, James Greig Smith, who held in the highest esteem the character and capabilities of him in whose honour the casket was designed.

He was one of the original members of the Medico-Chirurgical Society, and has frequently assisted in the work of the *Journal*. He declined to accept the office of President although invited to take the chair, but on his leaving Bristol he was offered and he accepted the only other distinction the Society could offer—that of honorary member.

Recently on the establishment of the University chairs he accepted the appointment of honorary Professor of Anthropology in the University of Bristol.

Although it is now twenty years since he retired from active medical life, meanwhile he has not been idle, for much of his time was occupied in literary works for various scientific journals. We have endeavoured to compile a list of the more important of these, more or less incomplete ; it forms a good memento of a life of great mental activity whose attainments were recognised by most of the Anthropological Societies of Europe.

It will be a source of gratification to Bristolians to be reminded that his portrait, painted by Miss Warne, and purchased by private subscription, is before us in the Bristol Room of the Municipal Art Gallery, where it will be a pleasing memorial of the charming personality of the original for many years to come.

One of his recent literary successes was the Long Fox Lecture, delivered in 1904, when he described and gave illustrations of " The Ideal Physician." The eulogy of his colleague and friend of many years may well be applied to himself as physician, ethnologist, anthropologist, archæologist, naturalist, for he was distinguished in all.

He lived to enjoy the reward which success in such aspirations seldom fails to bring, " Love, honour, and troops of friends." Love and honour remain, and will continue to remain so long as memory shall last.

### BIBLIOGRAPHY.

" On the Physical Characters of the Ancient and Modern Germans."—*Brit. Assoc. Rep.*, 1857 (pt. 2), pp. 118–20.

" On the Physical Character of the Natives of some parts of Italy and of the Austrian Dominions."—*Ethnol. Soc. Trans.*, 1859, i. 111.

" On the Physical Characteristics of the Jews."—*Ibid.*, p. 222.

"Sur la couleur des yeux et des cheveux des Irlandais."—Paris, *Anthrop. Soc. Bull.*, 1861, ii. 562–66.
"On the Supposed increasing Prevalence of Dark Hair in England."—*Anthropol. Rev.*, 1863, i. 310–12.
"On Hospital Dietaries."—*Dublin Quart. J. M. Sc.*, August, 1865.
"On the Testimony of Local Phenomena in the West of England to the Permanence of Anthropological Types."—*Anthropol. Soc. Mem.*, 1866, ii. 37–45.
"On the Head-forms of the West of England."—*Ibid.*, pp. 348–57.
"On the Physical Character of the Inhabitants of Bretagne."—*Ibid.*, 1870, iii. 359–65.
"On the Head-Form of the Danes."—*Ibid.*, pp. 378–83.
"On the Stature and Bulk of Man in the British Isles."—*Ibid.*, pp. 384–573.
"The Typical Races of Mankind."—*Proc. Bristol Nat. Soc.*, 1870, iv. 6–8.
"On the Anthropology of Lancashire."—*Brit. Assoc. Rep.*, 1870, xl. 143–4; *J. Anthropol.*, 1872, i. pp. xv–xviii.
"On the Ottoman Turks."—*Brit. Assoc. Rep.*, 1870, xl. 144.
"The Kelts of Ireland."—*J. Anthropol.*, 1870–71, 117–31.
"Address to the Department of Anthropology."—*Brit. Assoc. Rep.*, 1873, xliii. 134–40.
"Note on the Walloons."—*J. Anthropol.* 1873, ii. 18–20.
"On the Mortality of Adolescence."—*Brit. Assoc. Rep.*, 1875, 205.
"On the Death-rates of some Health Resorts, and specially of Clifton."—*Ibid.*, 1875, 205–6.
"On the Aborigines of Central Queensland."—*Anthropol. Inst. J.*, 1878, vii. 145–8.
*The Progress of Public Health in our own Times.*—8vo. Bristol. [1879.]
"On the Bulgarians."—*Anthropol. Inst. J.*, 1879, viii. 232–38.
"On Anthropological Colour Phenomena in Belgium and Elsewhere."—*Ibid.*, 1881, x. 374–9.
*Address on Health.*—8vo. London. 1880.
"On the Stature of the Inhabitants of Hungary."—*Ibid.*, 1882, xi. 410–13.
"Sur la couleur des cheveux et des yeux dans la France du nord et dans la France du centre."—Paris, *Soc. Anthropol. Bull.*, 1882, v. 146–59; 1883, vi. 683–4.
"English Surnames, from an Ethnological Point of View."—*Anthropol. Inst. J.*, 1883, xii. 231–41.
"The German and Rhætian Elements in Switzerland."—*Brit. Assoc. Rep.*, 1883, 574–5.
"Notice sur la couleur des cheveux et des yeux en Suisse."—*Neuchâtel, Soc. Sci. Bull.*, 1883, xiii. 203–213.
*Races of Britain.* 8vo. Bristol. 1885.
"The Physique of the Swiss as Influenced by Race and by Media."—*Brit. Assoc. Rep.*, 1888, 837–8.
"On Human Bones Discovered by General Pitt-Rivers at Woodcuts," etc. —*Ibid.*, p. 839.
"Observations on the Natural Colour of the Skin in certain Oriental Races."—*Ibid.*, 1889, 787.
*Rhind Lecture on Anthropological History in Europe.* Edinburgh. 1890.
"On the Dards and Siah-Posh Kafirs."—*Brit. Assoc. Rep.*, 1873, 901.
"On the Complexional Differences between Natives of Ireland with Indigenous and Exotic Surnames respectively."—*Ibid.*, 1894, 775.
"Physical Anthropology of the Isle of Man."—*Ibid.*, 1896, 925.
"On the Mediæval Population of Bristol."—*Ibid.*, 1898, 1013.
"Notes on Colour Selection in Man."—*Ibid.*, 1899, 876.
"On the Anthropology of West Yorkshire."—*Ibid.*, 1900, 902.
"On the Vagaries of the Kephalic Index."—*Ibid.*, p. 902.
"Long Fox Lecture: The Ideal Physician."—*Bristol M.-Chir. J.*, 1904.
"Huxley Lecture: Colour and Race in Europe."—*Anthropol. Inst. J.*, 1905.

